# Determination of Pb and Cd in Garlic Herb (Allium sativum) Planted in Gilan and Khuzestan Provinces Using Graphite Furnace Atomic Absorption Spectrometry

**Published:** 2012-05-28

**Authors:** Zahra Ramezani, Nasrin Aghel, Negar Amirabedin

**Affiliations:** 1 Toxicology Research Center, Ahvaz Jundishapur University of Medical Sciences, Ahvaz, IR Iran; 2 Plant Research Center, Faculty of Pharmacy, Jundishapur University of Medical Sciences, Ahvaz, IR Iran; 3 Faculty of Pharmacy, Jundishapur University of Medical Sciences, Ahvaz, IR Iran

**Keywords:** Garlic, Metals, Heavy

## Abstract

**Background:**

Foods are enriched with variety of chemical elements. Some of these elements are necessary for human health. These elements enter in liquid, the cells and other organs, certain amount of these elements are essential for body organs to work properly.

**Objectives:**

This research was conducted to compare the level of lead and cadmium contamination in garlic species planted in Ramhormoz and Rasht and discuss about the source of this contaminations.

**Materials and Methods:**

Twenty composite samples were collected from each province, Khozestan (Ramhormoz) and Gilan (Rasht). Each sample was burned according to instruction reported in AOAC. Then the white residue was dissolved in diluted nitric acid then the Pb and Cd contents were determined using graphite furnace atomic absorption spectrometer at 217 and 228.8 nm, respectively. No Pb level was detected in garlic cultured in both cities. The mean concentration of cadmium was 0.40 and 0.58 mg/kg in garlics planted in Ramhormoz and Rasht, respectively.

**Results:**

Statistical analysis showed significant difference between mean concentration of cadmium in Rasht garlic and the permitted value (*P* < 0.001). The cadmium level in Rasht garlic is much higher than the standard level.

**Conclusions:**

In order to test the reliability of the data obtained using this method, randomly selected samples were spiked with Pb and Cd standard solutions. The cadmium and lead recovery were reported 93.93 and 90.18 percent.

## 1. Background

Foods are enriched with variety of chemical elements. Some of these elements are necessary for human body. These elements are enter as liquid, cells and other body organs and perhaps the existence of certain amount of these elements are necessary for body organs to work properly. Lead and Cadmium are two unnecessary elements that accumulate the living organs for which these elements are toxic (1). Lead accumulates in the bodyand interferes in Vitamin D and Calcium metabolites; it is a neurotoxin whichcauses behavioral abnormalities (1). Cadmium ion are easily absorbed by vegetables and animal food distributed in kidney and liver consequents to many health problmes (2-4) it was shown that rice wheat, oyster, mussels and the kidney cortex of animals contain the highest amount of cadmium. (5, 6) Vegetables, especially leaf vegtables may contain elevated lead level when grown near sources of lead (7-11). vegetables grown in highly contaminated soils would obviously contain a high cadmium level. Hence determination of lead and cadmium contents of vegetables and complementary pharmaceutical is an important issue which attracts scientist's attentions all over the world (12-16). Even different reliable methods have been reported in the literature (17-19). Garlic is both a spicy food additive and herbal medicine. In Iran especially in North region, high amount of raw and cooked garlic is used daily. In result, the exposure incidence of these region population that leads to health hazards are significantly high. a periodical monitor of the extent of heavy metals contaminations in garlic, or further vegetables is mandatory.

## 2. Objectives

This research was conducted to compare the level of lead and cadmium contamination in garlic species planted in Ramhormoz and Rasht and discus about the sources of these contaminations.

## 3. Materials and Methods

All chemicals used in the present study, excluding Pb and Cd standard solutions, were analytical reagent grade and purchased from Merck, Germany. Stock solutions of 1000 μg.mL^-1^ of Pb^2+^ and Cd^2+^ were purchased from Chem Lab N.V., Belgium. GF/B filter papers were used for filtration purposes. Doubly distilled water was used in this study.

100 μg.mL^-1^ of lead and cadmium solution: 10 mL of lead and cadmium stock solutions were transferred to two seprate 100 mL volumetric flasks and disolved in distilled water. Working standard solutions identified in the instrument cook book and AOAC methods were prepared by successive dilutions of these intermediate solutions.

### 3.1. Sample Collection

Garlic samples were randomly collected in March and April 2007 from Rasht/Gilan and Ramhormoz/Khozestan farms to this purpose, 10 farms were randomly selected at six different locations in each agricultural area, and a total of 60 samples for each province were collected. Samples from each farm were divided into two group of three. Samples in each group were mixed together to compound composite samples. Consequently two composite samples from, each farm and an overall of 20 composite samples for each cultivated area were collected. The samples were dried in a clean dark place and were ground into powder. Powders were reserved in clean plastic vessels for further analysis.

### 3.2. Apparatus

Analytical Jena graphite furnace atomic absorption spectrometer (GFAAS), 5EA, Germany with D_2_ background correction was used for all assessments. Samples were burned in an Electrical Furnace EX 1200-6L (Iran). All glassware used in this experiment was soaked in 10% nitric acid for 24 hours, rinsed thoroughly in double distilled water, dried in an oven and cooled before use.

### 3.3. Sample Preparation

Samples were digested by the use of two following methods, dry ashing and wet digestion, which previously described in references 20 and 21. In wet digestion a precise amount of garlic powder (1g) were transferred to the Teflon beaker, 15ml of concentrated HNO_3_ and 5ml %40 HF were added and heated on a heater till no gas was evolved that was an indication of complete digestion (never let samples to become dry). Dry ashing was performed as described in AOAC (20). Briefly, before 2 g of sample powder were burned at 450°C for about 8 hours (a white ash was obtained). This residue dissolved in minimum amount of diluted HNO_3_. After filtration the resulting solution was transferred to 10 mL volumetric flask and diluted to the mark with diluted HNO_3_. This solution was immediately subjected to graphite furnace AAS analysis.

### 3.4. Graphite Furnace Atomic Absorption Determination

20 microliter of sample solution was transferred to the graphite tube then 5µl %1 orthophosphoric acid was added as modifier. Finally the program was run and the data was reported (20). The Pb and Cd analyzing Furnace programs are shown in (Table 1).

**Table 1:** Temperature Programs Set on the Graphite Furnace AAS for the Determination of Pb and Cd in Garlic Samples

Step	Pb a			Cd a		
	Temperature, °C	Ramp, Degree/S	Hold, S	Temperature, °C	Ramp, Degree/S	Hold, S
Drying1	105	7	25	105	5	10
Drying2	-	-	-	110	10	10
Pyrolysis	450	100	2	650	20	10
Auto Zero	450	0	4	650	0	4
Atomize	2000	2200	2	1300	400	5
Clean out	2500	1000	4	2500	200	3

^a^Abbreviations: Cd: Cadmium; Pb: Lead

### 3.5. Recovery

In order To determine the analysis accuracy, exact amounts of standards were added separately to two randomly selected samples prepared by dry ashing and wet digestion methods. The recovered standards were calculated from the difference and reported in (Table 2).

**Table 2:** Comparisons of Wet Digestion and Dry Ashing Reliability during Sample Preparation

Method	Metal	Standard Added, ng	Standard Recovered, ng	Recovered,%
Wet Digestion	Pb	17	4.27	25.11
	Cd	1	6.60	660.00
Dry Ashing	Pb	0.66	0.60	90.18
	Cd	0.50	0.47	93.93

## 4. Results

In order to select more reliable and easy digestion methods for preparing garlic samples two standard preparation methods (wet digestion and dry ashing) (20, 21) were selected, the accuracy of the data obtained by these two methods were evaluated by spiking certain amounts of standards in both prepared garlic samples. Table 2 showed that the extent of matrix effects were higher in wet digestion according to AOAC suggestion, dry ashing was chosen for the present study analyzes (20).

**Figure 1:**
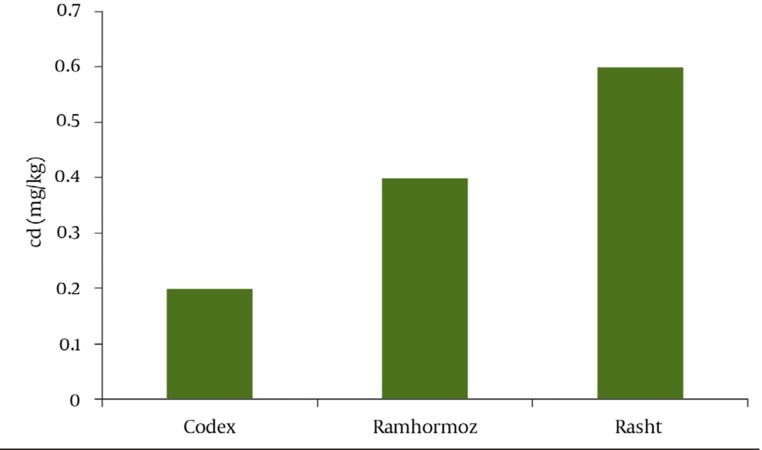
Graphical Comparison of Mean Concentration of Cadmium in Garlic Samples Collected from Ramhormoz and Rasht

The analysis results of 20 composite samples collected from the two cultivated areas shown in (Table 3)and confirmed the availability of No Pb. This is consistent with the results obtained from root vegetables by other researchers (13). the same results were obtained from onion samples in our research group (22). Formation of insoluble Pb compounds with some anions especially phosphate, immobilizes the metal in soil and water a (23, 24) and reduces lead bioavailability consequently vegetables mostly arecontaminated with atmosphere Pb. The Mean Cd contents of cultivated garlics in both provinces were higher than standard level of 0.2 mg/kg, reported by ***Codex*** (25) as it is graphically illustrated in (Figure 1). The mean Cd contents were compared with permitted level using student t-test. The results showed that there was a significant differences between cadmium contents of Rasht garlic and permitted value (*P* < 0.001), but there was no significant differences between those of Ramhormoz (*P* = 0.388). Students t-test also showed significant differences between cadmium contents of the both cultivated areas (*P* = 0.037). Consequently it was concluded that Rasht garlic is contaminated.

**Table 3:** Analysis Results of Cd Contents of Garlic Cultivated in Ramhormoz and Rasht (Results are Mean of Two Measurements)

Ramhormoz, mg/kg	Rasht, mg/kg
0.16	0.37
0	0.51
0.6	0.28
0.2	0.23
1.15	0.72
0.19	0.17
0.28	1.45
0.03	0.21
0.42	0.82
0.75	0.63
0.77	0.58
0.32	0.69
0	0.61
0.23	0.75
0	0.68
1.37	0.68
0.26	0.42
0.06	0.38
1.14	0.75
0.07	0.68
0.03 a	0.17 a
1.37 b	1.45 b
0.40 ± 0.38 c	0.58 ± 0.29 c

^a^Minimum

^b^Maximum

^c^Mean ± SD

## 5. Discussion

According to primary investigations of the regions, farmers commonly use organo phosphorous fertilizers, especially simple super phosphate and triple super phosphate. Fertilizers contain high amount of Cadmium and Lead. But Pb bioavailability reduced via complexion of pb ions by anions such as hydroxide, carbonate, sulphate, sulfide, and especially phosphates. In comparison to the Ramhormoz’s farmers, the farmers of Rash use uncontrolled amount of these fertilizers, which is closely related to the higher amount of Cd detected in the collected garlic samples. The results show the mean concentration of cadmium in Gilan garlic is higher than those of Khozestan. It may be due to higher oregano phosphorous fertilizer consumption rate. Since daily garlic consumption as food and medicine is routine in this regions, an alternative fertilizer with less hazards is of an essence. Soil heavy metal contamination and irrigation water are other concerns, which should be prevented by heavy metal analyzes and cultivated area controls before growing any plant.
